# Supplementary material for: Chemical Inhibition of Sterol Biosynthesis
Source: Biomolecules. 2024 Mar 28;14(4):410. doi: 10.3390/biom14040410 (PMC11048061; doi:10.3390/biom14040410)
Supplement: Supplementary file 1 [file biomolecules-14-00410-s001.zip › biomolecules-2910754-supplementary.pdf]

**Supplemental Table S1.** Databases and search strings used for initial literature search

| Database | Search String |
|---|---|
| EMBASE | (((('24 dehydrocholesterol reductase' OR dhcr24 OR '7 dehydrocholesterol reductase' OR dhcr7 OR 'sterol c5 desaturase' OR sc5d OR '3-β-hydroxysteroid-δ8-δ7-isomerase' OR 'emopamil-binding protein' OR ebp OR '14-dehydrocholesterol reductase' OR dhcr14 OR 'methyl sterol oxidase' OR sc4mol OR 'lanosterol synthase' OR LSS OR 'hydroxysteroid 17-beta dehydrogenase 7' OR hsd17b7 OR 'nad(p)h steroid dehydrogenase-like protein' OR nsdhl OR 'laminin b receptor*' OR lbr OR 'lanosterol 14α-demethylase' OR ldm) NEAR/4 (inhibit* OR antagon* OR block* OR 'irreversible bind*')):ab,kw,ti) OR (((reduce OR reduces OR reduced OR reducing OR low* OR depressed OR subnormal*) NEAR/5 ('24 dehydrocholesterol reductase' OR dhcr24 OR '7 dehydrocholesterol reductase' OR dhrc7 OR 'sterol c5 desaturase' OR sc5d OR '3-β-hydroxysteroid-δ8-δ7-isomerase' OR 'emopamil-binding protein' OR ebp OR '14-dehydrocholesterol reductase' OR dhcr14 OR 'methyl sterol oxidase' OR sc4mol OR 'lanosterol synthase' OR LSS OR 'hydroxysteroid 17-beta dehydrogenase 7' OR hsd17b7 OR 'nad(p)h steroid dehydrogenase-like protein' OR nsdhl OR 'laminin b receptor*' OR lbr OR 'lanosterol 14α-demethylase' OR ldm) NEAR/3 (activit* OR level* OR express* OR transcript* OR translat*)):ab,kw,ti)) AND ('drug dosage form comparison'/de OR 'drug dose comparison'/de OR 'phase 3 clinical trial topic'/de OR 'adverse drug reaction'/lnk OR 'drug administration'/lnk OR 'drug analysis'/lnk OR 'drug combination'/lnk OR 'drug comparison'/lnk OR 'drug concentration'/lnk OR 'drug development'/lnk OR 'drug dose'/lnk OR 'drug interaction'/lnk OR 'drug therapy'/lnk OR 'drug toxicity'/lnk OR 'intraperitoneal drug administration'/lnk OR 'intravenous drug administration'/lnk OR 'oral drug administration'/lnk OR 'pharmacokinetics'/lnk OR 'side effect'/lnk OR 'special situation for pharmacovigilance'/lnk OR 'subcutaneous drug administration'/lnk OR 'drug'/exp/mj OR drug:ab,kw,ti OR drugs:ab,kw,ti OR agent*:ab,kw,ti OR medicat*:ab,kw,ti OR chemotherap*:ab,kw,ti OR prescri*:ab,kw,ti OR pharm*:ab,kw,ti) |
| EBSCOhost MEDLINE | ( TI (((“24 dehydrocholesterol reductase” OR dhcr24 OR “7 dehydrocholesterol reductase” OR dhcr7 OR “sterol c5 desaturase” OR sc5d OR “3-β-hydroxysteroid-δ8-δ7-isomerase” OR “emopamil-binding protein” OR ebp OR “14-dehydrocholesterol reductase” OR dhcr14 OR “methyl sterol oxidase” OR sc4mol OR “lanosterol synthase” OR LSS OR “hydroxysteroid 17-beta dehydrogenase 7” OR hsd17b7 OR “nad(p)h steroid dehydrogenase-like protein” OR nsdhl OR “laminin b receptor*” OR lbr OR “lanosterol 14α-demethylase” OR ldm) N3 (inhibit* OR antagon* OR block* OR “irreversible bind*”)) OR ( (reduce OR reduces OR reduced OR reducing OR low* OR depressed OR subnormal*) N4 (“24 dehydrocholesterol reductase” OR dhcr24 OR “7 dehydrocholesterol reductase” OR dhcr7 OR “sterol c5 desaturase” OR sc5d OR “3-β-hydroxysteroid-δ8-δ7-isomerase” OR “emopamil-binding protein” OR ebp OR “14-dehydrocholesterol reductase” OR dhcr14 OR “methyl sterol oxidase” OR sc4mol OR “lanosterol synthase” OR LSS OR “hydroxysteroid 17-beta dehydrogenase 7” OR hsd17b7 OR “nad(p)h steroid dehydrogenase-like protein” OR nsdhl OR “laminin b receptor*” OR lbr OR “lanosterol 14α-demethylase” OR ldm) N2 (activit* OR level* OR express* OR transcript* OR translat*))) OR AB (((“24 dehydrocholesterol reductase” OR dhcr24 OR “7 dehydrocholesterol reductase” OR dhcr7 OR “sterol c5 desaturase” OR sc5d OR “3-β-hydroxysteroid-δ8-δ7-isomerase” OR “emopamil-binding protein” OR ebp OR “14-dehydrocholesterol reductase” OR dhcr14 OR “methyl sterol oxidase” OR sc4mol OR “lanosterol synthase” OR LSS OR “hydroxysteroid 17-beta dehydrogenase 7” OR hsd17b7 OR “nad(p)h steroid dehydrogenase-like protein” OR nsdhl OR “laminin b receptor*” OR lbr OR “lanosterol 14α-demethylase” OR ldm) N3 (inhibit* OR antagon* OR block* OR “irreversible bind*”)) OR ( (reduce OR reduces OR reduced OR reducing OR low* OR depressed OR subnormal*) N4 (“24 dehydrocholesterol reductase” OR dhcr24 OR “7 dehydrocholesterol reductase” OR dhrc7 OR “sterol c5 desaturase” OR sc5d OR “3-β-hydroxysteroid-δ8-δ7-isomerase” OR “emopamil-binding protein” OR ebp OR “14-dehydrocholesterol reductase” OR dhcr14 OR “methyl sterol oxidase” OR sc4mol OR “lanosterol synthase” OR LSS OR “hydroxysteroid 17-beta dehydrogenase 7” OR hsd17b7 OR “nad(p)h steroid dehydrogenase-like protein” OR nsdhl OR “laminin b receptor*” OR lbr OR “lanosterol 14α-demethylase” OR ldm) N2 (activit* OR level* OR express* OR transcript* OR translat*))) ) AND (MH "Drug Therapy+" OR TI (drug OR drugs OR agent* OR medicat* OR chemotherap* OR prescri* OR pharm*) OR AB (drug OR drugs OR agent* OR medicat* OR chemotherap* OR prescri* OR pharm*) OR MW (drug OR drugs OR agent* OR medicat* OR chemotherap* OR prescri* OR pharm*)) |
